# Sessile Serrated Lesions in Inflammatory Bowel Disease: Hidden Players in Colitis-Associated Colorectal Cancer?

**DOI:** 10.3390/jcm14228042

**Published:** 2025-11-13

**Authors:** Roberto de Sire, Diletta De Deo, Miriana Mercurio, Gianluca Franchellucci, Giulio Calabrese, Livio Bonacci, Mauro Sollai Pinna, Cristina Bezzio, Alessandro Armuzzi, Cesare Hassan, Alessandro Repici, Fabiana Castiglione, Sandro Ardizzone, Roberta Maselli

**Affiliations:** 1 Endoscopy Unit, Gastroenterology Department, Humanitas Research Hospital IRCCS, 20089 Rozzano, Italy; roberto.desire@humanitas.it (R.d.S.); diletta.dedeo@humanitas.it (D.D.D.); miriana.mercurio@humanitas.it (M.M.); gianluca.franchellucci@humanitas.it (G.F.); cesare.hassan@hunimed.eu (C.H.); alessandro.repici@hunimed.eu (A.R.); roberta.maselli@hunimed.eu (R.M.); 2 IBD Unit, Department of Clinical Medicine and Surgery, University Federico II, 80131 Naples, Italy; giuliocalabrese94@gmail.com (G.C.); liviobonacci@gmail.com (L.B.); fabcasti@unina.it (F.C.); 3 Department of Biomedical Sciences, Humanitas University, Pieve Emanuele, 20072 Milan, Italy; cristina.bezzio@hunimed.eu (C.B.); alessandro.armuzzi@hunimed.eu (A.A.); 4 Pathology Department, Humanitas Research Hospital IRCCS, 20089 Rozzano, Italy; mauro.sollai@humanitas.it; 5 IBD Unit, Gastroenterology Department, Humanitas Research Hospital IRCCS, 20089 Rozzano, Italy; 6 Endoscopy Unit, Gastroenterology Department, Humanitas San Pio X Hospital IRCCS, 20089 Rozzano, Italy

**Keywords:** Crohn’s disease, ulcerative colitis, sessile serrated lesion, traditional serrated adenoma, serrated epithelial change, visible dysplasia, colitis associated neoplasia, colorectal cancer, EMR, ESD

## Abstract

Sessile serrated lesions (SSLs) are well-known precursors of colorectal cancer in the general population, but their role in inflammatory bowel disease (IBD) is less clear. This narrative review summarizes what is known about the prevalence, molecular features, endoscopic detection, malignant potential, and management of SSLs in patients with IBD, highlighting where evidence supports action nowadays and where prospective studies are urgently needed. IBD-associated colorectal cancer has long been considered a consequence of the inflammation–dysplasia–carcinoma sequence, distinct from the conventional adenoma–carcinoma pathway. Increasing evidence, however, suggests that the serrated pathway, typically characterized by SSLs and traditional serrated adenomas (TSAs), may also contribute to IBD-related oncogenesis. This review synthesizes histopathological, molecular, endoscopic, and clinical data on SSLs in patients with IBD, with contextual reference to TSAs, sessile serrated lesions with dysplasia, and serrated epithelial change only when relevant to their interpretation or risk stratification. SSLs are now more frequently identified in IBD surveillance, especially in ulcerative colitis and the proximal colon, although prevalence estimates remain heterogeneous due to evolving definitions and significant interobserver variability. Molecular studies indicate that IBD-associated serrated lesions often harbor *BRAF* mutations but display a lower CpG island methylator phenotype than their sporadic counterparts, suggesting an inflammation-modified biology. While most hyperplastic polyps and non-dysplastic SSLs appear to pose limited neoplastic risk, dysplastic serrated lesions carry a markedly higher likelihood of synchronous or metachronous advanced neoplasia. Advances in high-definition endoscopy and chromoendoscopy improve the detection of these subtle, mucus-capped, flat lesions, while endoscopic resection is nowadays feasible in expert hands. Future priorities should include prospective multicenter cohorts integrating molecular profiling to refine surveillance strategies.

## 1. Introduction

Inflammatory bowel disease (IBD), comprising ulcerative colitis (UC) and Crohn’s disease (CD), is associated with an increased risk of colorectal cancer (CRC) [1,2,3].

This risk is particularly elevated in patients with long-standing, extensive UC or Crohn’s colitis (excluding isolated proctitis), with studies reporting a two- to three-fold increase compared to the general population, although absolute risks have declined with modern therapy. The magnitude of CRC risk in patients with IBD is shaped by several factors, including the extent and severity of colonic inflammation, disease duration, family history of CRC, concomitant primary sclerosing cholangitis (PSC), male sex, and younger age at IBD diagnosis [4]. Additional risk modifiers include features of a more aggressive disease course, such as high cumulative inflammatory burden, backwash ileitis, the presence of pseudopolyps, prior dysplasia, and colonic strictures [5].

Colorectal carcinogenesis in IBD diverges from the classical pathway observed in sporadic CRC (sCRC) [1]. While sCRC typically arises via the conventional adenoma–carcinoma sequence, IBD-associated CRC (IBD-CRC) is primarily driven by a chronic inflammation–dysplasia–carcinoma sequence, in which sustained mucosal injury and regeneration promote neoplastic transformation [6].

This inflammation-mediated pathway is characterized by oxidative stress, cytokine-induced DNA damage, and continuous epithelial turnover, often resulting in flat, nonpolypoid dysplasia rather than the polypoid adenomas common in sCRC. Molecular alterations in IBD-CRC include early *TP53* mutations, DNA aneuploidy, and less frequent *APC* and *KRAS* mutations, in contrast to sCRC, where *APC* loss is typically the initiating event, followed by *KRAS* activation, chromosomal instability (CIN), and late *TP53* loss [7,8,9,10].

These two distinct pathways currently represent the cornerstone of our understanding of CRC development in IBD. However, the potential involvement of the serrated neoplasia pathway, a well-established alternative route to CRC in the general population, remains poorly characterized in IBD. In non-IBD cohorts, serrated polyps (SPs), particularly sessile serrated lesions (SSLs), are recognized precursors of CRC, accounting for approximately 20–30% of sporadic CRCs [11]. While SSLs are increasingly reported in IBD patients, their true prevalence, histopathological characterization, and neoplastic potential remain unclear [12,13]. Moreover, chronic inflammation may obscure or mimic the morphology of SSLs, complicating recognition and classification.

Although this review primarily focuses on WHO-defined SSLs and SSL with dysplasia (SSL-D) in the setting of IBD [14], other serrated-appearing epithelial alterations occasionally described in IBD (e.g., serrated epithelial change, TSA-like dysplasia, hyperplastic-like change) are briefly addressed only when reported in the original studies and strictly for diagnostic context. These entities are not re-classified or interpreted as part of the serrated pathway unless supported by WHO criteria, in order to avoid conceptual ambiguity and ensure alignment with current consensus frameworks.

This narrative review focuses specifically on SSLs in IBD, examining available evidence on their histopathologic, molecular, and clinical features and their potential contribution to colitis-associated carcinogenesis. Other serrated patterns described in IBD are referenced only when necessary to provide context and avoid terminology overlaps, as the primary objective is to clarify the role and implications of SSLs in IBD rather than to comprehensively review all serrated subtypes.

## 2. Materials and Methods

### Search Strategy

This is a narrative review and does not follow systematic review methodology. We searched MEDLINE (via PubMed) and Embase from inception to 2025 using combinations of the following terms: “inflammatory bowel disease” OR “ulcerative colitis” OR “Crohn’s disease” AND “serrated” OR “sessile serrated” OR “SSL” OR “serrated epithelial change” AND “prevalence” OR “detection” OR “chromoendoscopy” OR “dysplasia” OR “neoplastic risk” OR “cancer”. We also screened guideline and consensus documents from ECCO, BSG, ESGE, ACG/AGA, and the USMSTF, and hand-searched reference lists of key articles. English-language studies were prioritized. Because this is not a systematic review, we favored higher-quality or more recent evidence (multicenter cohorts, guideline statements, mechanistic studies) and highlighted where data are extrapolated from non-IBD populations.

To maintain transparency despite the narrative nature of the review, we emphasize that article selection was based on clinical relevance, methodological contribution, and the ability to inform current understanding of serrated lesions in IBD. In areas where evidence is limited, selected small series and descriptive pathology studies were included, acknowledging that they do not meet strict systematic criteria but contribute meaningfully to this emerging field.

When extracting data, lesions were classified as WHO-defined SSL and SSL-D versus IBD-specific descriptive categories (e.g., “SSL-like dysplasia”, “TSA-like dysplasia”) exactly as labeled in the original articles, and these categories were analyzed separately to avoid terminological conflation.

Older studies used the terms “sessile serrated adenoma/polyps (SSA/Ps)” and “sessile serrated polyps (SSPs)” before the adoption of the WHO 2019 classification, and these do not always allow a clear distinction between nondysplastic SSL and SSL-D. For methodological rigor, lesions were recorded using the terminology reported in the original publications without retroactive re-classification.

As this is a narrative review, no formal study selection process was applied. Nevertheless, we preferentially included: (i) multicenter or population-based cohorts when available; (ii) consecutive surveillance series rather than selected case reports for prevalence estimates; (iii) studies with molecular or histologic correlation when discussing biology; and (iv) guideline or consensus documents when addressing management.

Taken together, these methods reflect a curated synthesis of the most relevant and influential literature, rather than a systematic or comprehensive literature review.

## 3. Histopathology and Molecular Features of SSLs

As SSLs are gaining increasing recognition in the field of IBD, a thorough understanding of their histopathological and molecular features, especially in comparison to their sporadic counterparts, is essential. Clarifying the similarities and differences between these lesion types may shed light on their respective biological behaviors, malignant potential, and implications for surveillance in the context of chronic inflammation.

In this review, SSL and SSL-D are used according to the World Health Organization (WHO) 2019 definitions [14]. By contrast, the label “SSL-like dysplasia” is not a WHO-defined entity, but a descriptive term used in IBD cohorts when serrated dysplasia resembles sporadic SSLs without fulfilling WHO criteria. In this manuscript, “SSL-like dysplasia” is therefore reported only when explicitly used in the original sources and is analyzed separately from WHO-defined SSL/SSL-D. Other serrated entities are mentioned only as reported in primary studies; historical terms (SSA/Ps, SSPs) are mapped to SSL for consistency. Serrated epithelial change (SEC) is described separately, as it represents an inflammation-associated serrated pattern that is not uniformly considered premalignant. SEC refers to a serrated epithelial pattern arising in chronically inflamed mucosa and is not included among WHO-defined serrated lesions. Its biological significance remains debated, and SEC is currently regarded as a morphologic reaction pattern rather than a formally recognized precursor lesion.

### 3.1. Sporadic Serrated Lesions

Historically, the SSLs were frequently under-recognized by endoscopists and often misclassified by pathologists as hyperplastic polyps (HPs), despite accumulating evidence of their malignant potential [13]. This diagnostic ambiguity stemmed from inconsistent terminology, evolving histopathological criteria, and limited understanding of their natural history. Notably, microvescicular HPs, a subtype of HPs, have historically represented a key source of diagnostic confusion, as they may display serrated crypt architecture and subtle basal crypt irregularities that overlap with early features of SSLs, thereby contributing to misclassification in routine practice [11]. The 2019 WHO classification clarified the SPs spectrum, now comprising HPs, SSLs, SSL-D, traditional serrated adenomas (TSAs), and unclassified serrated adenomas [14].

Among these, SSLs and TSAs are now established as true precursor lesions within the serrated pathway of colorectal carcinogenesis [11]. WHO 2019 defines SSLs by at least one unequivocal distorted crypt with basal dilatation, serrations to the base, horizontal growth along the muscularis mucosae, and asymmetric proliferation [14]. SSLs are predominantly located in the right colon, constitute 15–25% of SP and 4% of all colorectal polyps [15,16,17], and undergo dysplastic transformation (SSL-D) in 4–8%.

At the molecular level, sporadic SSLs follow a characteristic trajectory driven by activating mutations in *BRAF* (V600E, present in 70–80% of cases) or, less commonly, *KRAS* (about 9%), accompanied by extensive CpG island methylation (CIMP-high) [18]. CIMP-high promotes epigenetic silencing of tumor suppressor genes such as *MLH1* and p16^INK4a^, perturbing proliferation and differentiation, and promoting neoplastic progression. Notably, *MLH1* hypermethylation, detected in approximately 75% of SSL-D, drives progression to microsatellite instability–high (MSI-H) CRC, whereas SSL-D without *MLH1* silencing is more commonly associated with microsatellite stable (MSS) CRCs [19,20,21].

Transition to dysplasia is marked by *WNT* activation, via truncating mutations in *RNF43*, *APC*, or *ZNRF3*. This observation is supported by the presence of nuclear β-catenin accumulation and MYC overexpression in most SSL-D, but not in SSLs. MSI-H SSL-D may also carry mutations in additional *WNT* regulators such as *FBXW7*, *AXIN2*, and *MCC* [21]. In contrast to SSLs, TSAs constitute a rarer serrated subtype (<1% of colorectal polyps) but are likewise considered premalignant. They arise on *BRAF*- or *KRAS*-mutant backgrounds; *KRAS*-mutant TSAs are commonly CIMP-low (CIMP-L) and progress to *MSS* CRC. TSAs arising in the distal colon often display *SMOC1* gene methylation and silencing, molecular features linked to high-grade dysplasia and *MSS* CRC development [20,22,23]. Their *WNT* activation is usually driven by *PTPRK-RSPO3* fusions or *RNF43* mutations, not *APC* loss, underscoring their divergence from the conventional adenoma–carcinoma sequence [24].

### 3.2. Serrated Lesions in Inflammatory Bowel Disease

Although SSLs are now increasingly acknowledged within the neoplastic spectrum of IBD, their histological identification remains challenging due to chronic inflammation and regenerative mucosal changes that may mimic serrated features. In this review, the focus remains on WHO-defined SSL and SSL-D, as they currently represent the only serrated lesions with established neoplastic significance in IBD.

In the IBD series, the term “SSL-like dysplasia” is used descriptively and does not correspond to the WHO category of SSL/SSL-D. We therefore retain such terminology only when used by original authors, without re-classifying these lesions, and discuss them separately to avoid conflation with true SSL/SSL-D. Other serrated-appearing changes occasionally reported in IBD are also mentioned only when defined in source studies, to provide diagnostic context without implying established biological equivalence to sporadic counterparts [14].

In IBD patients, SPs are typically categorized histologically as SSL-like dysplasia, TSA-like dysplasia, and serrated dysplasia—not otherwise specified (SD-NOS). SSL-like and TSA-like subtypes are named for their morphological resemblance to their sporadic counterparts, whereas SD-NOS lacks defining features of either but exhibits complex serrated architecture with cytologic atypia [25,26,27].

HP–like change, although occasionally described in IBD mucosa, is generally interpreted as a reactive phenomenon rather than a neoplastic serrated pathway lesion and was therefore not further analyzed in this review [28].

Although SEC may superficially resemble hyperplastic HP-like change histologically, the distinction in IBD relies primarily on endoscopic and architectural features rather than isolated microscopic findings. HP-like change represents a reactive hyperplastic process in inflamed mucosa, whereas SEC manifests subtle but targeted endoscopic abnormalities, typically low-relief mucosal nodularity or plate-like changes, coupled with crypt disarray, serration irregularity, and architectural distortion (without basal dilatation or lateral growth patterns characteristic of SSLs) [27]. Thus, while histology alone may not always reliably differentiate SEC from reactive HP-like change, the combination of endoscopic context and architectural pattern remains critical for accurate classification [28].

In addition to these, the SEC has been described as a distinct morphological and molecular entity arising in chronically inflamed IBD mucosa and has been proposed as a potential precursor of IBD-CRC. Several cohorts report frequent early *TP53* mutations and clonal overlap with adjacent neoplasia, suggesting precursor potential, though progression data are inconsistent and SEC is not uniformly regarded as premalignant [28]. In a study by Parian et al., 8% of patients with SEC had synchronous dysplasia or adenocarcinoma, and 21% of those without prior neoplasia developed such lesions during follow-up [29]. SEC presents endoscopically as subtle, non-polypoid mucosal nodularity, often escaping detection during routine surveillance and typically discovered on random biopsies. Histologically, SEC is characterized by irregular crypt architecture, loss of perpendicular orientation to the muscularis mucosae, distorted serrations, and goblet cell-rich epithelium [25,30]. In contrast to SSLs, SEC lacks basal crypt dilatation and lateral expansion patterns (e.g., L-or bott-shaped crypts) [27].

Molecular profiling supports SEC as a genetically distinct lesion. In a cohort of 78 IBD patients, Ko et al. reported that SSLs, predominantly located in the proximal colon, frequently harbored *BRAF* mutations, while TSAs, more common in the distal colon of male patients, showed *KRAS* mutations [30]. In contrast, SECs exhibited a high prevalence of *TP53* mutations and a very low incidence of *BRAF* or *KRAS* alterations. A targeted next-generation sequencing (NGS) study further confirmed frequent *TP53* mutations in SECs, even in the absence of dysplasia or carcinoma, suggesting that SEC may represent an early event in the IBD-associated neoplastic sequence. Moreover, the detection of identical *TP53* mutations in SEC and adjacent neoplastic lesions supports a clonal relationship and reinforces its proposed role as a precursor of IBD-CRC [28].

While SSL-like dysplasias arising in the setting of IBD share several morphological and molecular features with sporadic SSLs, emerging evidence also highlights notable differences. A recent retrospective analysis [31] comparing serrated polyps from colitis-affected and unaffected mucosa found *BRAF* mutations in 75% of SSL-like dysplasias from inflamed segments and in 100% of SSLs from non-inflamed mucosa. *KRAS* mutations were identified in all TSA-like dysplasias and TSAs, irrespective of mucosal inflammation. Among SD-NOS lesions, 75% had *KRAS* and 25% *BRAF* mutations, reflecting their partial biological overlap with TSA-like dysplasia. These findings align with previous molecular studies [24,30]. Notably, the same retrospective study was the first to evaluate CIMP status in serrated lesions from patients with UC. CIMP positivity was found in 60% of TSA-like dysplasias, but in only 25% of SSL-like dysplasias and SD-NOS lesions from inflamed mucosa. This contrasts with sporadic serrated lesions, where CIMP is a hallmark of SSLs and generally rare in TSAs. The lower CIMP prevalence in SSL-like dysplasias within inflamed mucosa, alongside its unexpected presence in TSA-like dysplasias, suggests that chronic inflammation may modulate canonical serrated pathways [31].

**Figure 1:**
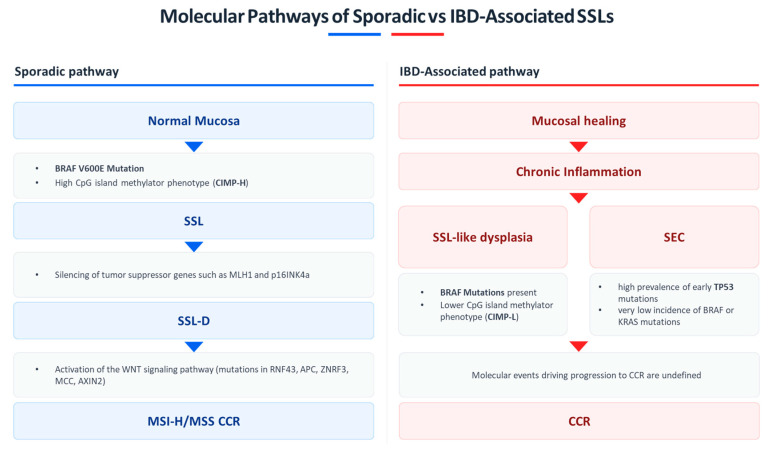
Schematic representation of the molecular pathways driving sporadic versus IBD-associated SSLs: In the majority of cases, the sporadic serrated pathway (left panel) is initiated by *BRAF* V600E mutation and a high CpG island methylator phenotype (CIMP-H), leading to epigenetic silencing of *MLH1* and p16^INK4a^, activation of the *WNT* signaling pathway, and progression from SSL to SSL-D and, when *MLH1* hypermethylation occurs, to microsatellite instability-high (MSI-H) CRC. The IBD-associated pathway (right panel) develops in the context of chronic mucosal inflammation, which may modify the serrated neoplastic process. SSL-like dysplasia frequently harbors *BRAF* mutations but exhibits a lower prevalence of CIMP (CIMP-L). Additionally, SEC may arise under chronic inflammatory stress, characterized by a high prevalence of early *TP53* mutations, potentially representing an alternative precursor to dysplasia and carcinoma in IBD. However, beyond these initial alterations, the molecular events driving progression to colorectal cancer in IBD-associated serrated lesions remain poorly characterized.

However, despite growing morphological and molecular data, the precise sequence of genetic and epigenetic events leading from SSL-like dysplasia to invasive carcinoma in the context of IBD remains unclear (Figure 1). Comparative molecular studies between sporadic and IBD-associated SSLs are needed to define their natural history, oncogenic potential, and clinical implications for surveillance in IBD populations.

## 4. Prevalence and Detection

### 4.1. Reported Rates in IBD Cohorts

While SSLs have been extensively characterized in the general population, data on their prevalence in patients with IBD remain limited and heterogeneous [32]. Published studies report both higher and lower rates of SSLs in IBD cohorts compared to non-IBD populations, reflecting a lack of consensus regarding their true frequency. Moreover, it remains unclear whether chronic inflammation facilitates the development of SSLs or whether their apparent prevalence is influenced by surveillance intensity and detection bias. The wide variability in reported rates likely stems from heterogeneity in study design, histopathological classification, endoscopic techniques, and patient characteristics [33]. This is partly because the current definition of SSLs goes back to the 2019 WHO classification.

Johnson et al. [32] and Lee et al. [33] have found it to be lower than the general, non-IBD population at 0.2–1.4%; however, these results were prior to the WHO definition alteration for SSL in 2019, and may have under-estimated the true prevalence of SSLs. A recent retrospective cohort study of a tertiary IBD center showed that SSLs, meeting the current definition according to the 2019 WHO classification, were detected in 7% of surveillance colonoscopies performed in IBD patients [34]. A retrospective cohort of 621 IBD patients found SPs in about one-third of cases, the majority being nondysplastic HPs or SSLs, while dysplastic SSLs and TSAs were uncommon. Their presence was independently associated with UC, male sex, and older age, suggesting that serrated lesions, mainly nondysplastic types, are relatively frequent in IBD, particularly in older men with UC [35]. In a single-center cohort of over 4200 IBD patients, SEC and SSPs were rare, with detection rates of 10/1000 and 2/1000, respectively. Given the low number of SSPs, analyses mainly focused on SEC, underscoring that both lesions are uncommon in IBD surveillance colonoscopy [32]. In a surveillance single-center cohort of 134 IBD patients, 147 SPs were detected, most being HPs or unclassifiable SPs. SSPs were less common but showed a clear predilection for the right colon compared with other serrated subtypes [36]. In another 14-year pathology study of over 6600 IBD patients, only 78 SPs were identified. HPs predominated, while SSLs and TSAs were uncommon and occurred more often in patients with UC [30].

### 4.2. Endoscopic Challenges

Surveillance colonoscopy is a mainstay of IBD management, as early detection and removal of neoplastic lesions reduces CRC-related morbidity and mortality. In light of this, guidelines recommend surveillance colonoscopy beginning 8–10 years after disease onset [37] or sometimes earlier (after 6–8 years) [38], given reports of CRC arising within the first decade, with intervals tailored to risk (annual for PSC or strictures; 2–3 yearly for post-inflammatory polyps or family history; every 5 years in low-risk cases) [39]. However, guidelines focus on conventional dysplasia and do not explicitly address the detection or management of SSLs in the context of IBD. Surveillance methodology has evolved substantially with the advent of advanced imaging. Historically, standard-definition white-light endoscopy (SD-WLE) plus random 4-quadrant biopsies every 10 cm was standard, based on the assumption that most dysplasia was “invisible” with targeted visible lesions [40]. However, SD-WLE was limited by poor image quality and often failed to reveal subtle mucosal changes, leading to high miss rates. Dye-based chromoendoscopy (DCE) improved dysplasia detection and was endorsed by the 2015 SCENIC consensus as the preferred modality through targeted biopsies and outperformed SD-WLE [41].

More recently, the introduction of high-definition white-light endoscopy (HD-WLE) improved imaging resolution with enhanced mucosal detail, allowing for more effective lesion recognition without dye application [42]. In parallel, virtual chromoendoscopy (VCE), a dye-free digital contrast enhancement technique, has gained prominence for its ease of use, efficiency, and comparable performance in identifying dysplasia. By simply pushing a button, VCE provides an instant digital staining, enhancing colonic mucosal details and vascularization [43].

Advances in endoscopic imaging have shifted surveillance in IBD away from random biopsies toward targeted detection. The role of non-targeted sampling is increasingly questioned, with growing evidence supporting advanced imaging as the new standard. [44].

Within this evolving landscape, SSLs represent a particularly difficult target. Several studies have demonstrated that SSLs are missed far more often than conventional adenomas. In a randomized trial, up to 80% of SSLs in the proximal colon were overlooked during the first examination and detected only on a second inspection [45]. Similarly, an observational study in which trainee-performed colonoscopies were immediately repeated by an expert endoscopist found an SSL miss rate of 85.7% [46]. While these results come from non-IBD populations, they highlight how easily SSLs can escape detection. In IBD, where inflammation, scarring, pseudopolyps and architectural distortion may further obscure subtle serrated features, detection is likely to be even more difficult than in the general population [47]. Yet, robust data in this setting are lacking, and this knowledge gap underscores the need for dedicated studies to quantify SSL miss rates in the setting of IBD surveillance [48].

SSLs typically appear as flat or slightly elevated pale lesions, often covered by a mucus cap with indistinct borders, frequently located in the proximal colon, and can be easily missed during standard white-light endoscopy [49,50].

In IBD, they may be further obscured, emphasizing the importance of advanced imaging. DCE with indigo carmine or methylene blue remains most sensitive for flat lesions, while VCE (e.g., NBI, iScan) provides additional detail but requires expertise and optimal bowel preparation [51]. Suspicious features, mucus cap, ill-defined borders, vascular irregularity, should trigger careful assessment, ideally by experienced endoscopists. Accurate identification of dysplasia, now including recently described, hard-to-recognize non-conventional patterns, is therefore a critical and challenging task for pathologists interpreting IBD mucosal biopsies [40].

Given the diagnostic uncertainty and increased risk of neoplasia in longstanding IBD, any suspicious lesion, particularly in the proximal colon, should be carefully examined, and in cases of uncertainty, assessment by an experienced endoscopist is recommended. The presence of a mucus cap, indistinct borders, or abnormal vascular pattern should raise suspicion for SSLs.

Notably, although the adoption of advanced imaging is shifting IBD surveillance away from random biopsies toward targeted inspection, management decisions in patients with IBD still do not rely solely on endoscopically visible lesions. Histologically detected “invisible dysplasia” continues to carry its prognostic and therapeutic implications and is managed accordingly in current IBD practice. This reflects the central role of histology, independent of endoscopic visibility, in guiding treatment and surveillance decisions in IBD-associated neoplasia [40,52,53].

**Figure 2:**
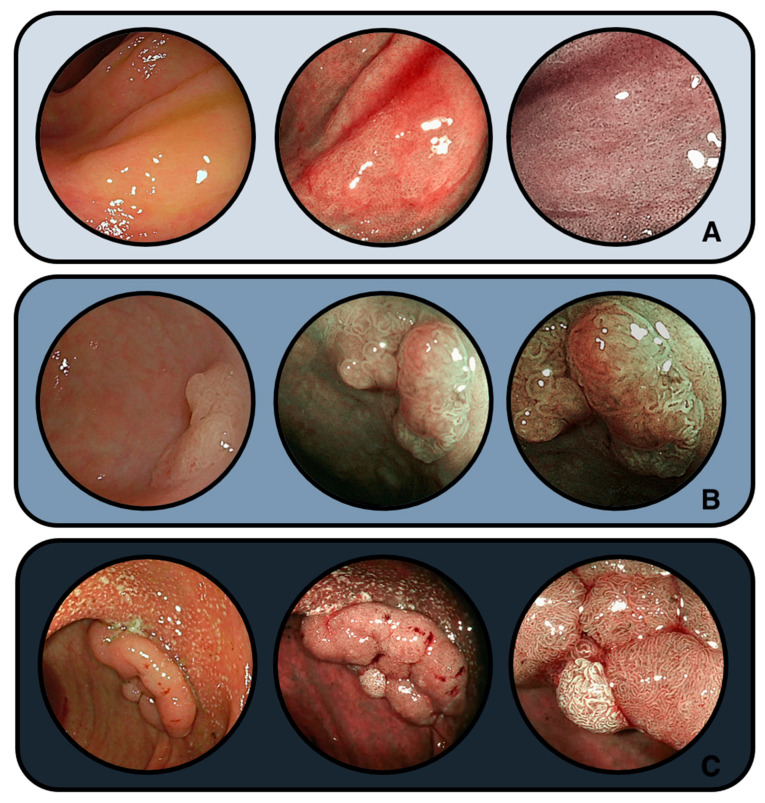
Imaging with HD-WLE and blue light imaging (BLI) plus focal interrogation of different types of visible colitis-associated nonpolypoid lesions: (**A**) SSL without dysplasia; (**B**) SSL with dysplasia; (**C**) tubulovillous adenoma (low and high-grade dysplasia).

**Figure 3:**
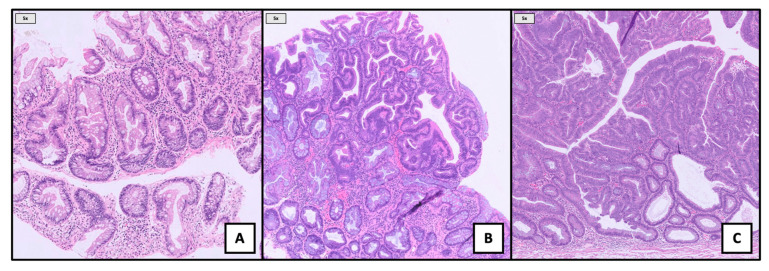
Histologic features (5×, H&E) of different types of visible colitis-associated nonpolypoid lesions after resection: SSL without dysplasia (**A**); SSL with dysplasia (**B**); and tubulovillous adenoma with low and high-grade dysplasia (**C**).

Representative endoscopic appearances of colitis-associated serrated lesions are illustrated in Figure 2, highlighting their morphological continuum from nondysplastic SSLs to dysplastic lesions and adenomas. Histological features are represented in Figure 3.

### 4.3. Differences by IBD Subtype

Several retrospective studies have investigated the prevalence and characteristics of SPs in IBD, with particular attention to differences between UC and CD, while other series compared serrated lesions with conventional adenomatous dysplasia.

In a Japanese cohort of more than 2000 UC patients, SPs were detected in 1.8% of cases, predominantly in men with extensive, long-standing colitis and persistent inflammation. Among 26 lesions located in colitis-affected segments, most displayed SSL-like dysplasia (58%), typically proximal and associated with *BRAF* mutations, whereas TSA-like dysplasia was more distal and linked to *KRAS* mutations [31]. In a retrospective cohort of 621 IBD patients, SPs were found in 32%. These included 92 HPs, 88 nondysplastic SSLs, 13 dysplastic SSLs, and 5 TSAs. Independent predictors for the presence of SPs were UC (OR 1.77), male sex, and increasing age [35]. In a single-center retrospective study of 83 IBD patients, 96 adenomatous dysplasias, 25 SSA/Ps, and 4 TSAs were identified (prevalences: 4.95%, 1.39%, and 0.31%, respectively). Serrated lesions were significantly more common in females (*p =* 0.046). While no differences were observed between UC and CD, anatomical distribution varied: adenomatous dysplasia was more distal, SSA/Ps more proximal, and TSAs evenly distributed. In CD, this pattern was highly significant, while in UC, lesions were more evenly distributed throughout the colon [33]. Finally, in an analysis of 65 serrated lesions from 39 IBD patients, UC accounted for the vast majority of cases (82% UC, 18% CD), with lesions predominantly polypoid and located in the left colon. Histologically, 21 were SSLs without dysplasia, 2 with dysplasia, 9 TSAs, 28 HPs, and 5 inflammatory pseudopolyps. Molecular analysis revealed a sidedness pattern similar to sporadic disease: *BRAF* mutations in right-sided and *KRAS* in left-sided lesions (*p* < 0.001) [54].

Collectively, SPs are more frequently encountered in UC, particularly in long-standing and extensive disease. UC-associated lesions often display SSL-like morphology and show molecular features (*BRAF* mutation in proximal or *KRAS* in distal colon) comparable to sporadic counterparts. In contrast, CD patients appear to harbor fewer SPs, with a distribution and phenotype closer to the general population, suggesting potential differences in the pathways of colorectal carcinogenesis between UC and CD.

## 5. Neoplastic Potential and Risk of Progression

CRC is one of the most severe long-term complications of colonic IBD. Although its incidence has declined over the past two decades, likely due to improved therapies and surveillance [55], UC and colonic CD patients still have more than double the relative risk compared with the general population [56,57].

IBD-related CRC accounts for about 2% of annual CRC mortality and 10–15% of IBD deaths, with higher mortality (HR 1.45) and worse 5-year survival in patients under 50 years compared with sCRC [58]. While the absolute risk remains relatively low, estimated at 1.1–5.4% after 20 years of disease, the likelihood in individual patients is influenced by factors such as disease extent, cumulative inflammatory burden, sex, and age at onset [52].

To mitigate this risk, IBD patients are routinely enrolled in surveillance colonoscopy programs aimed at detecting and removing precancerous lesions. In the general population, serrated lesions are thought to account for up to 25–30% of sCRC, primarily through the sessile serrated pathway [59,60]. Recognition of these lesions was historically poor, particularly before 2010, when most were misclassified as HPs [61].

However, growing evidence since then has demonstrated that individuals with SPs, particularly SSLs and TSAs, may carry a 2- to 4-fold increased risk of developing synchronous or metachronous advanced CRN, including high-grade dysplasia (HGD) and invasive cancer [62]. Despite this, the malignant potential of SPs in the context of IBD remains incompletely defined. To date, only a limited number of small, retrospective studies have explored the risk of CRN in IBD patients harboring serrated lesions, highlighting the need for further investigation in this unique clinical setting [30,36].

### 5.1. Evidence Linking SSLs to Dysplasia/Carcinoma in IBD

In recent years, interest in a serrated neoplastic pathway in IBD has increased, as several studies have reported features in IBD-associated SSLs that parallel those seen in sporadic serrated lesions. In particular, *BRAF* mutations and CpG island methylator phenotype (CIMP), together with loss of MLH1 in dysplastic SSLs, have been described in IBD cohorts [27,31], suggesting that a serrated route to CRC may operate alongside the conventional adenoma–carcinoma sequence, especially in long-standing colitis [59].

When assessing neoplastic risk in IBD, only dysplastic SSLs and TSAs were significantly linked to subsequent advanced CRN (HR 13.51, 95% CI 3.11–58.68, *p* < 0.001). In contrast, HPs (HR 1.98, *p* = 0.36) and nondysplastic SSLs (HR 0.87, *p* = 0.89) were not associated with increased risk. Since 91% of SPs in the cohort fell into these low-risk categories, the findings suggest limited malignant potential for most serrated lesions in IBD. The substantially higher risk in the small subset of dysplastic SSLs and TSAs (9%) highlights the need for precise histological classification to guide risk stratification [35].

In summary, while serrated lesions are well established as precursors of sCRC, their contribution to colitis-associated carcinogenesis appears confined to a minority of dysplastic SSLs and TSAs. Clarifying their true malignant potential in IBD remains a critical unmet need.

### 5.2. Evidence from Surveillance Cohorts

Although prospective registries are lacking and many published data derive from small retrospective cohorts, several large consecutive surveillance cohorts and population-based studies have also assessed serrated lesions in IBD and provide essential context for risk interpretation. To avoid overstating early evidence, historical case reports and small descriptive series are cited to contextualize initial observations, whereas conclusions rely primarily on larger surveillance cohorts and population-level datasets.

These include the nationwide population-based cohort by Axelrad et al. [63], the ulcerative-colitis–specific consecutive series by Nishio et al. [31], and the multicenter cohort focused on dysplastic serrated lesions by de Jong et al. [35], all of which have reported non-incidental rates of serrated lesions and a risk signal concentrated in dysplastic subtypes. This section does not aim to provide a systematic synthesis, but rather to summarize key surveillance evidence most relevant to current clinical decision making.

Several surveillance studies report a relatively low but non-negligible prevalence of SSLs in IBD, particularly in colonic segments affected by chronic inflammation. A limited number of cases have demonstrated progression from SSLs to dysplasia or carcinoma, although such transformations appear to be rare. For instance, a few case reports have documented high-grade dysplasia or invasive carcinoma arising within SSLs, especially in patients with concurrent risk factors such as PSC, extensive colitis, or a history of dysplasia. However, long-term follow-up data remain sparse, and most cohort studies lack sufficient power to quantify the risk of progression with confidence [64].

Although most SPs detected in IBD patients are considered low risk, the same cohort study demonstrated that a minority of lesions, specifically SSLs with dysplasia and TSAs, were significantly associated with the development of subsequent advanced CRN (HR 13.51, *p* < 0.001). In contrast, HPs and nondysplastic SSLs carried no measurable increase in CRN risk (HR 1.98 and 0.87, respectively; *p* > 0.3). These results highlight the importance of histological subtyping in risk stratification, as only a small fraction of serrated lesions may have meaningful neoplastic potential in the IBD population [35].

A large nationwide study of 41,880 IBD patients found an increased risk of neoplastic colorectal polyps (aHR 1.23), with the highest risks seen for SSPs (aHR 8.50) and traditional serrated adenomas (aHR 1.72). Risk was greater in UC than CD, especially with early-onset IBD and longer disease duration [63]. In a UC-specific series from Japan, serrated lesions were present in 1.8% of UC patients and accounted for 14% of neoplastic lesions, illustrating that although SPs may be relatively uncommon in UC, they contribute significantly to overall neoplasia in this subgroup [31]. In a retrospective single-center cohort (2006–2012) among 4208 IBD individuals, patients with SEC had a cumulative incidence of subsequent CRN of 12% at 1 year and 30% at 3 years, compared with 4% and 9%, respectively, in matched controls (*p* = 0.047). This association was not statistically significant after adjustment for prior or synchronous dysplasia (*p* = 0.09) [32]. Jackson et al., in a retrospective single-center surveillance cohort of 134 IBD patients, observed that synchronous multifocal visible dysplasia occurred significantly more often in patients with SSPs or serrated polyp unclassified (SPU) (44.5% and 66%) compared to those with HP (12%; *p* = 0.031).

Among 13 IBD patients with an index SSPs followed for a median of 6 years, 61.5% developed metachronous visible dysplasia or additional SSPs. Larger index SSPs carried a higher risk: each additional millimeter increased the risk of subsequent visible dysplasia by ~10% (HR 1.1; *p* = 0.028) [36]. Ko et al., in a retrospective single-center pathology study, showed SSLs were frequently associated with *BRAF* mutations and an increased prevalence of synchronous or metachronous dysplasia compared to HPs, suggesting possible progression along a serrated pathway in IBD. Although hazard ratios were not specified, the molecular features supported a neoplastic potential distinct from that of HPs [30]. PSC-associated IBD is a distinct subgroup in which nearly one-third of patients develop dysplasia, reflecting a markedly higher neoplastic risk than IBD alone. In one study, PSC-IBD-associated dysplasia was more often non-conventional (61% vs. 25%, *p* < 0.001), endoscopically invisible (66% vs. 21%, *p* < 0.001), and right-sided (59% vs. 47%, *p* = 0.045), with the rate of advanced neoplasia almost twice that seen in non-PSC IBD patients (37% vs. 22%, *p* = 0.035) [65].

Overall, although retrospective cohorts provide some evidence that dysplastic serrated lesions contribute meaningfully to neoplastic progression in IBD, the overall risk remains difficult to quantify. The heterogeneity of published data highlights substantial knowledge gaps that continue to hamper risk stratification and inform the ongoing debate on how serrated lesions should be managed within IBD surveillance frameworks.

### 5.3. Malignant Potential: Established Risks and Remaining Gaps

Despite the suggestive evidence, the true malignant potential of SSLs in IBD remains uncertain. Dysplastic serrated lesions have been consistently associated with an increased risk of advanced neoplasia in IBD, whereas the malignant potential of non-dysplastic serrated lesions remains less clearly defined. Histological diagnosis is often challenging due to background inflammation, architectural distortion, and interobserver variability, which complicates lesion classification and risk stratification [66,67].

Furthermore, many SSLs may be indolent or biologically distinct from their sporadic counterparts, especially in the inflamed mucosal environment of IBD. There is currently no consensus on whether SSLs in IBD should be managed more aggressively than conventional serrated lesions, nor are there standardized guidelines regarding their surveillance or resection. The literature on IBD-related serrated lesions is limited to small case series and studies that often label lesions as “SSL-like” or “TSA-like” due to diagnostic ambiguity, which further underscores uncertainty regarding their natural history and malignant risk [25,66]. In contrast to the hypothesis that chronic inflammation promotes serrated neoplasia in patients with IBD, a retrospective multicenter study found that the risk of metachronous advanced neoplasia following index SP detection was similar between patients with and without IBD. No significant differences were observed in time from IBD diagnosis to polyp detection, treatment intensity, inflammatory markers, or mucosal inflammation (all p > 0.05). Importantly, SPs occurred independently of inflammation, suggesting that IBD status alone may not necessitate altered surveillance intervals in patients with serrated lesions [68].

This ambiguity underscores the need for larger, high-quality prospective studies with molecular correlates to clarify the natural history and risk of malignant transformation of SSLs in IBD. Finally, expert societies recognize the potential neoplastic risk of serrated lesions but highlight the lack of standardized guidelines for SSL management, specifically in IBD [69].

## 6. Surveillance and Management Implications

The management of CRN in IBD patients has evolved substantially over the past two decades, largely driven by improvements in endoscopic technology and a better understanding of dysplasia biology. The advent of HD-WLE, DCE, and VCE has shifted practice from reliance on random biopsies toward targeted inspection and resection of visible lesions, thereby reducing sampling error and improving dysplasia detection [70].

Despite these advances have reshaped the surveillance paradigm, no specific recommendations exist for SSLs in patients with IBD. While major guidelines acknowledge SSLs as part of the neoplastic spectrum, they do not define tailored surveillance intervals [40,52,53]. Meanwhile, the European Society of Gastrointestinal Endoscopy (ESGE) has issued relevant documents, including performance measures for IBD colonoscopy, which set standards for mucosal inspection and reporting [41], and the 2024 ESGE guideline on polypectomy and endoscopic mucosal resection, which outlines detailed strategies for the resection of SPs in the general population. Although these recommendations are often extrapolated to IBD practice, they have not yet been formally validated in this specific setting [71].

Taken together, these observations demonstrate that SSLs are acknowledged across modern surveillance guidelines but are not yet assigned specific surveillance intervals or management algorithms in IBD.

### 6.1. Are SSLs in IBD Addressed by Current Guidelines?

Current surveillance strategies for IBD-associated neoplasia derive from international guidelines, which recommend initiating colonoscopy 8–10 years after diagnosis of extensive colitis, with intervals tailored to risk factors such as inflammatory burden, prior dysplasia, and concomitant PSC [52].

The SCENIC consensus in 2015 represented a turning point by endorsing HD-WLE as standard, recommending DCE where expertise is available, and emphasizing resection of visible lesions. Importantly, this document predated the WHO 2019 serrated classification and therefore did not specifically identify SSLs as distinct precursors; serrated morphology would likely have been subsumed under “flat dysplasia” [40].

The ECCO 2023 guideline acknowledges SSLs but manages them within the broad category of visible dysplasia: if resection is complete (R0), the surveillance schedule reverts to their baseline IBD risk profile; SSLs without dysplasia and located outside inflamed segments should be managed as sporadic lesions, given the low associated risk of advanced neoplasia. ECCO does not provide separate intervals for serrated lesions and openly notes the absence of evidence to guide SSL-specific pathways [52].

The updated BSG guideline introduced a structured risk stratification (high-, intermediate-, low-) categories, with annual surveillance for patients with PSC, previous dysplasia, or extensive pseudopolyposis, and 3–5 yearly surveillance for lower-risk groups. SSLs are recognized within the dysplastic spectrum but are not considered an independent risk factor warranting shorter intervals [40]. Similarly, the comprehensive 2025 BSG guideline reiterates the importance of serrated lesions but provides no specific recommendations [72].

In addition, the ESGE performance measures for colonoscopy in IBD emphasize complete documentation, adequate withdrawal time, and meticulous inspection, noting the importance of detecting subtle lesions such as SSLs, but do not offer SSL-specific metrics [72].

Thus, across all major societies, SSLs are recognized, but management remains embedded in conventional dysplasia pathways. To provide a clearer overview, the major international guidelines addressing CRC surveillance in IBD have been summarized in Table 1, presented in chronological order. However, the detection of SSLs within segments previously or currently affected by colitis, especially those with architectural or cytological atypia, requires nuanced interpretation.

### 6.2. Endoscopic Resection Strategies

Compared with sporadic counterparts, SSLs arising in colitis segments pose distinctive endoscopic challenges; successful resection hinges on advanced imaging and careful technique. These lesions can be easily overlooked, particularly in the context of pseudopolyps, scarring, or active colitis. High-definition imaging, complemented by DCE or VCE, is critical to enhance lesion contrast, improve detection, and guide safe resection [70].

Resection strategies depend on lesion size: cold snare polypectomy with a 1–2 mm margin achieves > 95% complete resection for SSLs ≤ 9 mm; endoscopic mucosal resection (EMR) or underwater EMR (U-EMR) are preferred for 10–19 mm lesions; piecemeal EMR remains standard for ≥20 mm, though endoscopic submucosal dissection (ESD) may be considered in selected expert centers when en bloc R0 resection is essential for accurate histological staging in case of suspected dysplasia [73].

Importantly, piecemeal resection of large, serrated lesions is associated with local recurrence rates of up to 15–20% in non-IBD cohorts, a concern that is plausibly amplified in the inflamed and fibrotic mucosa of IBD. For this reason, a site check within 6 months after piecemeal EMR of SSLs in IBD, mirroring recommendations in the general population [71]. The approach to resection of SSLs within colitis-affected segments must balance lesion morphology, size, location, and local expertise. En-bloc resection with negative (R0) margins is the standard of care, as R1 resection R1 is associated with significantly higher recurrence and neoplastic progression [74].

The management of visible lesions in patients with IBD has evolved with the advancement of endoscopic resection (ER) techniques, such as ESD and EMR. Several recent studies have evaluated the safety, efficacy, and outcomes of these procedures in IBD patients, particularly in the context of UC. While both techniques show promising results, their effectiveness can vary depending on lesion size, morphology, and the presence of submucosal fibrosis [73].

Hirai et al. retrospectively analyzed 238 lesions in UC patients. Among these, 142 were treated with EMR (including 22 SSLs) and 96 with ESD (including 12 SSLs), showing low recurrence (2.7%) and metachronous neoplasia in 6.1%. After a median follow-up of 34.7 months, all five reported deaths occurred in the surgery group. Overall survival was significantly higher in the ER group (*p =* 0.0085), supporting ER as a viable option in selected UC patients, with careful surveillance for metachronous lesions [75].

Iacopini et al. prospectively evaluated 9 long-standing UC patients with large (>20 mm) superficial non-polypoid neoplasms, mostly laterally spreading non-granular tumors (LST-NG). ESD was performed en bloc with R0 resection in 8 lesions and was curative in 7, including one sessile serrated adenoma. No invisible dysplasia or cancer was detected during a median 24-month follow-up. ESD can achieve curative resection in IBD, but it is technically challenging due to frequent submucosal fibrosis, underscoring the need for careful patient selection and strict long-term surveillance [76].

A retrospective study by Kasuga et al. analyzed 11 lesions from 9 UC patients treated with ESD, including one SSL, compared to 19 lesions from 9 patients who underwent colectomy. The en bloc and curative resection rates for ESD were 91% and 82%, respectively. ESD is a feasible and effective approach for large visible lesions in UC, though technical difficulties may arise in cases with endoscopic signs of fibrosis or scarring [77].

Ngamruengphong et al. retrospectively studied 45 dysplastic colorectal lesions in 41 IBD patients, including 4 serrated adenomas/polyps. En bloc and R0 resection rates were 96% and 76%, respectively. Over a median follow-up of 18 months, local recurrence was 2.6%, while metachronous lesions were identified in 31%. The study concluded that ESD, when performed by experts, is a safe and effective treatment for large dysplastic lesions in IBD [78].

Nishio et al. compared EMR (63 lesions) and ESD (39 lesions) in 102 colorectal tumors from 74 patients with UC, showing a higher R0 resection rate with ESD (97%) compared to EMR (80%), particularly for 11–20 mm lesions (94% vs. 55%) and non-polypoid tumors (100% vs. 65%). Perforations occurred in 4 ESD cases and metachronous HGD developed in 3 patients during follow-up. Of the lesions, 19 (19%) were SPs, including SSL and serrated adenomas, with 10 treated by EMR and 9 by ESD. The findings support ESD for ≥11 mm and non-polypoid lesions, while EMR may be suitable for ≤10 mm polypoid lesions [79].

Kaltenbach et al. retrospectively included 326 IBD patients with 63 lesions, identifying 14 cases of SSLs. ER of nonpolypoid colorectal lesions was found to be feasible (with a success rate of 96.8%) and associated with a low incidence of adverse events (AEs) of 1.5%. Moreover, in this study, the authors provide valuable long-term outcome data, demonstrating a low rate of recurrence (6.3%; 95% CI, 1.8–15.5) [80].

More recently, a retrospective Italian multicenter study included 91 IBD patients (82% with UC) with 96 high-risk colorectal advanced neoplasms (HR-CANs), 14.6% of which were SSLs. ESD or hybrid ESD (h-ESD) was performed in 82.3% and 17.7% of cases, achieving en bloc, R0, and curative resection rates of 95.8%, 85.4%, and 83.3%, respectively. After a mean follow-up of 23.4 months, local recurrence and metachronous lesions each occurred in 3.1%, with 11.5% requiring surgery post-resection, positioning ESD as an effective and safe treatment for HR-CANs in IBD patients, with favorable medium- and long-term outcomes [81].

Table 2 shows a summary of published studies evaluating ER (including EMR and ESD) for dysplastic and serrated lesions in IBD.

Recent advances in techniques and devices, such as traction-assisted [82,83] and underwater ESD [84], as well as thin-needle knives with high-pressure waterjet [85], have enhanced the feasibility, efficacy, and safety of colorectal ESD. These innovations may be particularly valuable in IBD, where submucosal fibrosis and adipose-rich tissue often complicate resection. They support the role of ESD in carefully selected IBD patients, although procedures should be centralized in expert referral centers. Importantly, pre-resection biopsy should be avoided unless there is a strong suspicion of invasive cancer, as it may induce fibrosis and hinder subsequent excision.

### 6.3. Post-Resection Surveillance and Uncertainties

Post-resection surveillance of SSLs in IBD remains an area of uncertainty, as no major guideline currently provides lesion-specific intervals distinct from conventional dysplasia management.

Both the ECCO 2023 malignancy guideline and the 2025 BSG surveillance update recommend that patients return to risk-stratified IBD surveillance schedules following complete resection (R0) of visible lesions, regardless of serrated morphology [52,53]. In practice, clinicians individualize follow-up when serrated lesions contain dysplasia or require piecemeal removal, reflecting concerns extrapolated from sporadic cohorts.

Evidence from non-IBD populations has consistently demonstrated increased recurrence risk after piecemeal EMR of large SSLs, with rates approaching 15–20% at first follow-up, prompting international societies to recommend site checks at 6–12 months [71,86]. Real-world series in IBD suggest that the same principle holds true: Jackson et al. reported that patients with SSLs frequently developed metachronous dysplasia during surveillance, particularly when index lesions were large or removed piecemeal [36]. Similarly, Ko et al. observed that SPs in IBD were often associated with synchronous or subsequent conventional dysplasia, reinforcing the rationale for closer interval monitoring [30].

Despite these observations, the malignant potential of nondysplastic SSLs in IBD remains debated. Notably, a contemporary multicenter cohort found that SPs in patients with IBD carried a metachronous neoplasia risk comparable to that observed in non-IBD populations, arguing against automatically shortening surveillance solely on the basis of IBD status when serrated lesions are present [68]. By contrast, risk appears concentrated in the subset of dysplastic serrated lesions (SSL-D or TSA), which were independently associated with subsequent advanced neoplasia in an IBD cohort [35], in line with earlier single-center data indicating a higher burden of synchronous/metachronous dysplasia around SSLs [36].

Overall, current practice reflects a pragmatic compromise. SSLs without dysplasia that are completely resected are generally followed within baseline IBD surveillance schedules, whereas SSL-D, TSA, or piecemeal-resected large SSLs typically prompt an early site check at 6 months before resumption of standard risk-based intervals. Whether this strategy adequately mitigates risk in the IBD population is unknown, underscoring the need for dedicated prospective cohorts and molecular correlates to inform future guidelines [75].

Taken together, while SSLs are increasingly recognized during IBD surveillance, current guidelines continue to subsume them under conventional dysplasia pathways without providing lesion-specific intervals. Resection strategies are largely extrapolated from the general population, with cold snare, EMR, or U-EMR applied according to size, and site checks recommended after piecemeal resections or when dysplasia is present [36]. The absence of IBD-specific prospective data leaves major uncertainties regarding malignant potential, recurrence risk, and optimal follow-up, underscoring the need for dedicated studies to determine whether SSLs should be considered independent risk modifiers in future surveillance algorithms [52,53].

## 7. Knowledge Gaps

Despite growing attention to serrated biology in IBD, the clinical significance of SSLs remains unsettled. Most evidence comes from small, retrospective series conducted under varying diagnostic criteria, leading to heterogeneous estimates of prevalence and progression. As a result, management is largely extrapolated from sporadic serrated pathways, while uncertainty persists due to inconsistent definitions and limited long-term outcome data [69,87].

### Diagnostic Variability and Its Implications for Surveillance Practice

SSLs are notoriously difficult to identify, even in non-IBD populations, because of their intrinsic morphology. In IBD, chronic inflammation, pseudopolyps, and scarring further obscure recognition. Endoscopically, this increases the likelihood of missed or incompletely resected lesions, while histologically, regenerative crypt distortion can mimic serrated architecture [30,88].

Distinguishing SEC from true serrated dysplasia can be challenging due to overlapping histologic features and substantial interobserver variability. SEC, while not currently considered premalignant, particularly when lacking cytologic atypia, may occasionally demonstrate hypermucinous or villiform architecture that warrants MDT review and expert colonoscopic re-evaluation. SSLs with definitive dysplasia, particularly in the setting of chronic inflammation, may represent a form of non-conventional dysplasia and necessitate complete ER [32].

In a multicenter survey conducted through telepathology, biopsy specimens from 30 colonoscopies in 20 patients were reviewed by 20 gastrointestinal pathologists in a blinded fashion. Serrated features were identified only rarely, being present in 7 biopsies from endoscopically visible lesions in 4 patients, 2 of which showed low-grade dysplasia (LGD) and 2 HGD. These findings illustrate the intrinsic difficulty of recognizing serrated patterns in the IBD setting, where the absence of standardized diagnostic criteria fosters variability and subjective interpretation, raising concerns about reproducibility and clinical reliability [89].

On histologic examination, regenerative changes frequently mimic serrated crypt architecture, leading to high interobserver variability when distinguishing SSL-like dysplasia from reactive serration. Multicenter reviews emphasize these diagnostic challenges and underscore the need for consensus definitions [27]. In addition, SEC remains a particular challenge: while some interpret it as a reactive phenomenon, molecular studies have shown *TP53* mutations and clonal overlap with adjacent neoplasia, supporting its potential role as a precursor lesion [28]. Others, however, have reported weaker or confounded associations, underscoring the need for standardized criteria and reporting [26,90]. Overall, diagnostic reproducibility remains limited, underscoring the need for consensus definitions, standardized criteria, and expert pathology input in IBD-associated serrated lesions.

Given the well-documented interobserver variability in IBD-associated dysplasia diagnosis, histologic evaluation should be performed by expert gastrointestinal pathologists, and a second review is recommended when feasible. Evidence from reproducibility studies demonstrates that agreement improves substantially when cases are reviewed by specialized pathologists or in consensus settings [25,66,89].

Artificial intelligence (AI) has been explored only as a research adjunct in this context, mainly through computer-aided diagnosis (CADx). Early studies suggest potential diagnostic utility, but no AI system has been validated for routine use in IBD-associated serrated lesions and current evidence remains preliminary [91].

On the molecular level, most studies have focused on *BRAF* and *KRAS* mutations. Other mechanisms, such as aneuploidy, mismatch repair status, and epigenetic signatures, remain underexplored. Flow cytometric studies have identified aneuploid subsets of SEC and TSA-like lesions, but outcome correlations are lacking [92]. Without prospective registries with standardized histology, molecular profiling, and longitudinal follow-up, the natural history of IBD-associated serrated lesions will remain speculative.

Beyond diagnostic variability, current surveillance guidelines manage serrated lesions within the broader framework of visible dysplasia, with decisions driven by resectability and dysplasia grade rather than serrated subtype [52,53].

In practice, many clinicians extrapolate from sporadic serrated pathways, recommending early site checks after piecemeal EMR or dysplastic SSLs, while returning small nondysplastic lesions to standard intervals [93].

Operational barriers compound this variability: uptake of DCE remains inconsistent, training in serrated detection is limited, and advanced pathology tools (digital pathology, molecular adjuncts) are not widely available [94]. Technical challenges, flat morphology, indistinct margins, and background inflammation underscore the importance of high-quality colonoscopy and careful histopathologic evaluation to minimize the risk of interval cancers.

Separately from pathology applications, AI has also been explored in the endoscopic setting for IBD-associated neoplasia. Abdelrahim et al. developed a deep learning system (RetinaNet/ResNet-101) that correctly detected and characterized several neoplastic lesions, including eight SSLs [95]. However, these findings are exploratory and require large-scale validation before any clinical applicability can be assumed, particularly for serrated lesions in the setting of IBD surveillance.

**Figure 4:**
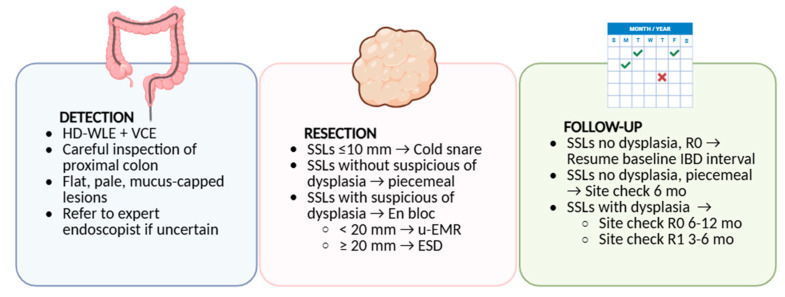
Clinician-facing algorithm for the management of visible SSLs in IBD. The algorithm highlights key steps in detection (high-definition endoscopy—HD-WLE, virtual chromoendoscopy—VCE, careful proximal colon inspection), resection (cold snare for small lesions, EMR or ESD for larger or complex lesions), and follow-up (intervals tailored to histology, completeness of resection, and patient-level risk factors). While largely extrapolated from non-IBD populations, these principles may guide individualized management until dedicated evidence becomes available.

To translate current evidence into clinical practice, we propose a pragmatic algorithm outlining the detection, resection, and follow-up of SSLs in IBD patients (Figure 4). These diagnostic and operational gaps reinforce the need for high-quality colonoscopy, expert pathology input and multidisciplinary review when managing serrated lesions in IBD, in order to minimize the risk of interval cancers.

## 8. Conclusions and Future Directions

The recognition of serrated lesions in the context of IBD has expanded our understanding of colorectal carcinogenesis beyond the conventional adenoma–carcinoma sequence. Current evidence indicates that SPs occur more frequently in UC than in CD, particularly in patients with extensive, long-standing, and active disease. Histologically, SSL-like dysplasia predominates, often proximal and *BRAF*-mutated, whereas TSA-like lesions are more distal and *KRAS*-driven. Although overall prevalence remains low, these findings suggest that serrated pathways may contribute to cancer development in IBD and should be integrated into risk assessment models.

From a clinical perspective, SSLs should be considered within surveillance and management frameworks, with emphasis on complete resection, careful histology, and tailored follow-up for dysplastic or piecemeal-resected lesions. Trials and surveillance algorithm validation are required to assess whether endoscopic detection of serrated lesions should modify surveillance intervals, and how these findings should be integrated into existing dysplasia management pathways.

Accurate histologic assessment remains a cornerstone of care, and evaluation by expert gastrointestinal pathologists, ideally with dual review, is recommended given the known variability in diagnosing IBD-associated dysplasia.

Future research should prioritize clarification of the natural history of serrated lesions in patients with IBD, including their progression risk and interplay with chronic inflammation. Molecular profiling studies are needed to determine whether UC- and CD-associated serrated lesions share the same oncogenic drivers as sporadic counterparts or display IBD-specific signatures. Establishing prospective registries will be crucial for capturing incidence, outcomes, and management strategies in real-world populations.

## Figures and Tables

**Table 1:** Surveillance and management recommendations for SSLs in IBD across major guidelines.

Guideline (Year)	Primary Focus	Surveillance Policy in IBD	Resection/Management of Visible Lesions	Position on SSLs in IBD and Post-resection Follow-Up
SCENIC Consensus (2015) [40]	Dysplasia surveillance & management in IBD; imaging standards	Begin 8–10 years after extensive colitis; risk-stratified thereafter; prefer HD-WLE and DCE in expert settings	Resection of all visible lesions; colectomy for unresectable or flat/invisible dysplasia	SSLs not addressed (pre-WHO 2019); no SSL-specific intervals
ESGE Performance Measures (2022) [41]	Quality standards for colonoscopy in IBD	Not interval-focused; emphasizes HD imaging, complete mucosal inspection, documentation, service-level QA	Not a resection guideline	Highlights need to detect subtle/flat lesions (incl. serrated) but no SSL-specific metrics or intervals
ECCO Guidelines (2023) [52]	IBD & malignancies	Intervals based on extent, cumulative inflammatory burden, PSC, prior dysplasia; MDT review	Visible dysplasia resect if complete (R0) is feasible; management individualized	SSLs acknowledged within dysplasia spectrum; after R0, surveillance reverts to baseline IBD risk; no SSL-specific intervals; evidence gap noted
ESGE Polypectomy/EMR Update (2024) [71]	Technical guidance for resection (general population)	Not IBD-specific for intervals; advises site-check after piecemeal EMR	SSL ≤ 9 mm: cold snare with 1–2 mm margin; 10–19 mm: EMR/U-EMR; ≥20 mm: piecemeal EMR; selective ESD in expert centers	Provides detailed SSL resection strategy; commonly extrapolated to IBD; pragmatic 6–12 months site-check after piecemeal or dysplasia-containing lesions
BSG Surveillance Guideline (2025) [53]	Colorectal surveillance framework in IBD	Structured risk tiers (e.g., annual for high-risk such as PSC/previous dysplasia; 3–5 years for lower-risk categories) with QA metrics	Prefer HD imaging; resect visible lesions when feasible; MDT pathways	SSLs acknowledged but not an independent risk modifier; no SSL-specific intervals after complete resection
BSG IBD Guideline (2025) [72]	Comprehensive adult IBD management (incl. malignancy risk)	Adopts risk-stratified surveillance as above	MDT-based management of dysplasia	Notes serrated lesions but no tailored SSL algorithms

**Table 2:** Studies evaluating endoscopic resection of dysplastic and serrated lesions in IBD. SSL-specific endoscopic outcome data are limited due to low lesion prevalence in IBD. Studies included here evaluate resection outcomes in IBD-associated dysplasia and serrated lesions; SSL-specific details are reported when available.

First Author	Study Design	Population	Intervention	Key Outcomes	Follow-Up
Hirai et al. [75]	Retrospective multicenter	UC, 238 colorectal lesions	EMR (142, incl. 22 SSL); ESD (96, incl. 12 SSL)	Perforation 2.5% (higher in ESD: 6.3%); recurrence 2.7%; metachronous neoplasia 6.1%; OS higher in ER group	Median 34.7 mo
Iacopini et al. [76]	Prospective multicenter case series	UC, 9 pts with large (>20 mm) lesions	ESD en bloc in 8/9; curative in 7 (incl. 1 SSL)	No invisible dysplasia/cancer; technically challenging due to fibrosis	Median 24 mo
Kasuga et al. [77]	Single-center retrospective	UC, 9 pts, 11 lesions	ESD (incl. 1 SSL); compared to colectomy (19 lesions)	En bloc 91%; curative 82%; feasible despite fibrosis/scarring	Median 25 mo
Ngamruengphong et al. [78]	Retrospective multicenter	IBD, 41 pts, 45 lesions	ESD (incl. 4 serrated adenomas/polyps)	En bloc 96%; R0 76%; 1 perforation (2.4%); recurrence 2.6%; metachronous 31%	Median 18 mo
Nishio et al. [79]	Retrospective single-center	UC, 74 pts, 102 lesions	ESD (39 lesions); EMR (63 lesions); 19 SPs	R0: ESD 97% vs. EMR 80%; 4 perforations; no recurrence; metachronous HGD in 3 pts	Mean 12 mo
Kaltenbach et al. [80]	Retrospective multicenter	IBD, 326 pts; 63 nonpolypoid lesions	ER (EMR/ESD/standard); 14 SSL	Success 96.8%; AE 1.5%; recurrence 6.3%	Mean 14.1 ± 26.1 mo
Maselli et al. [81]	Retrospective multicenter	IBD, 91 pts; 96 HR-CANs (14.6% SSL)	ESD (82.3%) or h-ESD (17.7%)	En bloc 95.8%; R0 85.4%; curative 83.3%; AE 12.5%; recurrence 3.1%; metachronous 3.1%	Mean 23.4 mo

## Data Availability

Not applicable.

## Abbreviations

The following abbreviations are used in this manuscript:

ACG	American College of Gastroenterology
AE	Adverse event
AGA	American Gastroenterological Association
AI	Artificial intelligence
APC	Adenomatous polyposis coli (gene)
AXIN2	Axis inhibition protein 2 (gene)
BLI	Blue light imaging
BRAF	v-RAF murine sarcoma viral oncogene homolog B1 (gene)
BSG	British Society of Gastroenterology
CE	Chromoendoscopy
CD	Crohn’s disease
CIN	Chromosomal instability
CIMP	CpG island methylator phenotype
CIMP-H	CpG island methylator phenotype–high
CIMP-L	CpG island methylator phenotype–low
CRC	Colorectal cancer
CRN	Colorectal neoplasia
DCE	Dye-based chromoendoscopy
ECCO	European Crohn’s and Colitis Organisation
EMR	Endoscopic mucosal resection
ER	Endoscopic resection
ESGE	European Society of Gastrointestinal Endoscopy
ESD	Endoscopic submucosal dissection
FBXW7	F-box/WD repeat-containing protein 7 (gene)
HD-WLE	High-definition white-light endoscopy
HGD	High-grade dysplasia
HP	Hyperplastic polyp
HR-CAN	High-risk colorectal advanced neoplasia
IBD	Inflammatory bowel disease
IBD-CRC	IBD-associated colorectal cancer
LGD	Low-grade dysplasia
LST-NG	Laterally spreading tumor—non-granular type
MCC	Mutated in colorectal cancers (gene)
MDT	Multidisciplinary team
MLH1	MutL homolog 1 (gene)
Mo	months
MSI-H	Microsatellite instability–high
MSS	Microsatellite stable
NBI	Narrow band imaging
NGS	Next-generation sequencing
NOS	Not otherwise specified
OS	Overall survival
PSC	Primary sclerosing cholangitis
Pts	patients
QA	Quality assurance
R0	Complete (negative-margin) resection
R1	Incomplete (positive-margin) resection
RNF43	Ring Finger Protein 43 (gene)
SCENIC	Surveillance for Colorectal Endoscopic Neoplasia Detection and Management in IBD Consensus
SEC	Serrated epithelial change
SD-NOS	Serrated dysplasia—not otherwise specified
sCRC	Sporadic colorectal cancer
SMOC1	SPARC-related modular calcium-binding protein 1 (gene)
SP	Serrated polyp
SPU	Serrated polyp, unclassified
SSA/P	Sessile serrated adenoma/polyp (historical term, mapped as SSL)
SSP	Sessile serrated polyp (historical term, mapped as SSL)
SSL	Sessile serrated lesion
SSL-D	Sessile serrated lesion with dysplasia
TSA	Traditional serrated adenoma
UC	Ulcerative colitis
U-EMR	Underwater endoscopic mucosal resection
U-ESD	Underwater endoscopic submucosal dissection
VCE	Virtual chromoendoscopy
WHO	World Health Organization
WNT	Wingless/integrated signaling pathway
ZNRF3	Zinc and ring finger 3 (gene)
